# Anorexia of ageing: a key component in the pathogenesis of both sarcopenia and cachexia

**DOI:** 10.1002/jcsm.12192

**Published:** 2017-04-27

**Authors:** John E. Morley

**Affiliations:** ^1^ Division of Geriatric Medicine Saint Louis University School of Medicine 1402 S. Grand Blvd., M238 St. Louis MO 63104 USA

**Keywords:** Anorexia, Aging, Cachexia, Sarcopenia

## Abstract

The anorexia of aging was first recognized as a physiological syndrome 30 years ago. Its major causes are an alteration in fundal compliance with an increase in antral stretch and enhanced cholecystokinin activity leading to increased satiation.This anorexia leads to weight loss in aging persons and is one of the component causes of the aging related sarcopenia. This physiological anorexia also increases the risk of more severe anorexia when an older person has an increase in inflammatory cytokines such as occurs when they have an illness. This results in an increase in the anorexia due to cachexia in older persons.

Anorexia is an important component of the cachexia syndrome^1^, ^2^ and also plays a role in the pathogenesis of sarcopenia.^3^, ^4^, ^5^ In a community study, anorexia was shown to be independently associated with sarcopenia.^6^ With ageing, there is a decrease in food intake known as the anorexia of ageing coupled with a decline in muscle mass and an increase in fat mass.^7^, ^8^, ^9^ The protective effect of obesity, especially when an older person becomes ill, is well recognized—the obesity paradox.^10^, ^11^, ^12^, ^13^

The physiological anorexia of ageing places the older person at increased risk of severe anorexia and weight loss when they develop an illness associated with an increased in inflammatory cytokines or an increase in tumours producing lactate.^14^, ^15^, ^16^, ^17^ There are multiple causes of the anorexia of ageing (*Figure* 1).^18^, ^19^, ^20^ Declining smell and taste plays a minor role in the decreased food intake. Changes in compliance of the fundus of the stomach due to nitric oxide deficiency and decreased antral stretch play a major role in postprandial anorexia, as does delayed gastric emptying in response to large meals.^21^, ^22^, ^23^ Because of this, there is an increase in food intake when liquid dietary supplements are used rather than solid food.^24^ Cholecystokinin (CCK) is the major gastrointestinal satiety hormone.^25^ CCK levels increase with ageing, and CCK is a more effective satiety agent with ageing.^26^, ^27^ Other gut satiety hormones like gastrin‐releasing peptide/bombesin, glucagon‐like peptide 1 and amylin do not appear to change much with ageing.^28^, ^29^ Leptin, a hormone produced by adipose cells, increases with increased fat mass and appears to play a role in the anorexia of ageing.^30^, ^31^ Hypertriglyceridemia blocks the ability of leptin to cross the blood–brain barrier.^32^ Male hypogonadism leads to an increase in leptin.^33^ The effects of ageing on ghrelin are controversial.^34^ The ghrelin analogue, anamorelin, is a potent enhancer of food intake.^35^, ^36^

**Figure 1:**
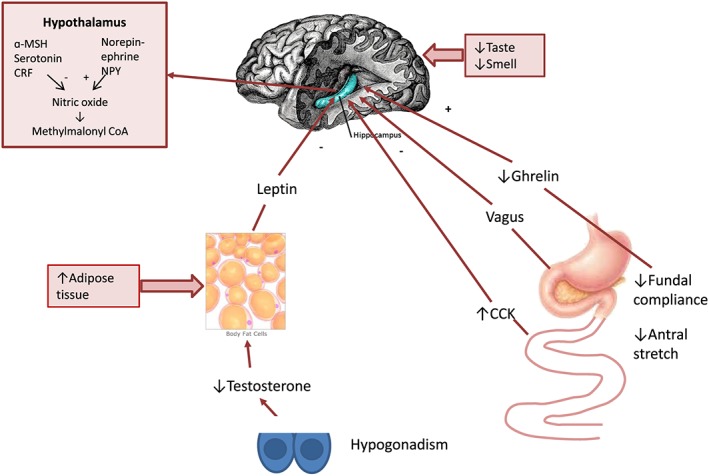
Simplified diagram of factors involved in the pathogenesis of the anorexia of ageing. CRF, corticotrophin releasing hormone; NPY, neuropeptide Y; MSH, melanocyte‐stimulating hormone; CCK, cholecystokinin.

The central regulation of feeding is a very complicated process.^37^ Multiple monoamines (especially serotonin and norepinephrine) and neuropeptides (e.g. neuropeptide Y, melanocortin, corticotrophin‐releasing factor) converge on the nitric oxide/methylmalnyl coenzyme A system to modulate food intake.^16^, ^37^, ^38^, ^39^, ^40^ Serotonin is a particularly anorectic agent, and in cancer, the effect of serotonin is potentiated.^41^ Ghrelin produces its effects by stimulating nitric oxide synthase.^42^ The ghrelin agonist, GHRP‐2, has been shown to increase food intake in persons with anorexia nervosa.^43^ Lactate, which is elevated in many cancers, has a direct inhibitory effect on methylmalonyl coenzyme A.^16^

Sarcopenia is defined as the decline in function due to the loss of muscle mass.^44^, ^45^ There are multiple causes of sarcopenia.^46^ The age‐related anorexia decreases muscle mass, and this can be aggravated by low grade production of inflammatory cytokines in chronic disease.^6^, ^47^

Persons with the anorexia of ageing are at risk of developing severe anorexia when exposed to high levels of inflammatory cytokines as occurs in the anorexia–cachexia syndrome.^48^, ^49^, ^50^ Persons with illnesses are apt to develop depression with an increase in the anorectic neurotransmitters, serotonin and corticotrophin‐releasing factor.^37^, ^38^

The data presented here support the concept that the anorexia of ageing is a major risk factor for older persons developing sarcopenia and/or cachexia. In addition, weight loss together with sarcopenia are major causes of the physical frailty syndrome.^51^, ^52^, ^53^, ^54^ For these reasons, we strongly recommend regularly monitoring and treating nutritional abnormalities in older persons.^55^, ^56^, ^57^, ^58^ When anorexia is associated with weight loss, the appropriate nutritional supplement is a leucine‐enriched essential amino acid mixture.^59^, ^60^ Drugs such as dronabinol and megestrol acetate have a small effect in increasing food intake.^61^, ^62^ Other drugs are under development to increase food intake and/or decrease muscle wasting.^63^

## Conflict of interest

The author has no conflict of interest regarding this work.
